# Polylactide-Grafted Metal-Alginate Aerogels

**DOI:** 10.3390/polym14061254

**Published:** 2022-03-21

**Authors:** Grigorios Raptopoulos, Ioannis Choinopoulos, Filippos Kontoes-Georgoudakis, Patrina Paraskevopoulou

**Affiliations:** 1 Inorganic Chemistry Laboratory, Department of Chemistry, National and Kapodistrian University of Athens, Panepistimiopolis Zografou, 15771 Athens, Greece; 2 Industrial Chemistry Laboratory, Department of Chemistry, National and Kapodistrian University of Athens, Panepistimiopolis Zografou, 15771 Athens, Greece; ichoinop@chem.uoa.gr (I.C.); fkontoes@chem.uoa.gr (F.K.-G.)

**Keywords:** aerogels, alginate, polylactide, polylactide-g-alginate

## Abstract

Τhis work describes the synthesis of PLA-grafted M-alginate (g-M-alginate; M: Ca^2+^, Co^2+^, Ni^2+^, Cu^2+^) aerogels. *DL*-lactide (LA) was attached on the surface of preformed M-alginate beads and was polymerized, using stannous octoate as catalyst and the –OH groups of the alginate backbone as initiators/points of attachment. The material properties of g-M-alginate aerogels were not affected much by grafting, because the linear PLA chains grew on the M-alginate framework like a brush and did not bridge their points of attachment as in polyurea-crosslinked M-alginate aerogels. Thus, all g-M-alginate aerogels retained the fibrous morphology of their parent M-alginate aerogels, and they were lightweight (bulk densities up to 0.24 g cm^−3^), macroporous/mesoporous materials with high porosities (up to 96% *v*/*v*). The BET surface areas were in the range of 154–542 m^2^ g^−1^, depending on the metal, the nature of the alginate framework and the PLA content. The latter was found at about 15% *w*/*w* for Ca- and Ni-based materials and at about 29% *w*/*w* for Co- and Cu-based materials. Overall, we have demonstrated a new methodology for the functionalization of alginate aerogels that opens the way to the synthesis of polylactide-crosslinked alginate aerogels with the use of multifunctional monomers.

## 1. Introduction

Aerogels are the lightest known solid materials, consisting of solid colloidal or polymeric networks expanded throughout their entire volume by a gas, like air, hence the name “aerogel” [1,2]. The first aerogels were based on inorganic oxides, biopolymers and proteins [3,4,5], but among them silica aerogels stood out and were more extensively studied and exploited commercially. In later years, the development of aerogels expanded to most classes of chemical compounds, e.g., inorganic materials (oxides, carbides, ceramics, chalcogenides and metals), organic polymers or biopolymers, hybrid organic-inorganic aerogel composites and carbon-based aerogels [6].

Among biopolymer-based aerogels, those based on alginates are particularly attractive because they provide metal-doped aerogels that can be easily synthesized from inexpensive and environmentally non-hazardous starting materials [7,8,9,10,11,12,13], and because they are also carbonizable [14]. Their synthesis involves gelation of sodium alginate with divalent (e.g., Ca^2+^, Ba^2+^, Co^2+^, Ni^2+^, Cu^2+^ or Zn^2+^) or trivalent (e.g., Fe^3+^ or Al^3+^) cations, which bind to the carboxylate groups of different polymer chains, with the entire process taking place in aqueous solutions [11,15,16].

Biomedical applications, including wound dressings, drug delivery and antimicrobial activity, and environmental applications, including water decontamination and gas sorption, are major application fields for alginate and alginate-based aerogels, not only because of their biocompatibility and easy gelation but also because of the vast range of ions that can be used for gelation [17,18,19]. A drawback is their limited stability in physiological/natural conditions, first because they are mechanically weak materials collapsing in water, and second because they can exchange gelation cations with monovalent cations (e.g., Na^+^) present in the surrounding media and, therefore, dissolve [20,21]. In order to address these issues and improve the properties/applications of alginate aerogels, several types of composite materials have been reported in the literature.

One approach in this direction is through the polymer-crosslinking technology, which was initially developed for inorganic aerogels [22,23,24,25,26]. In this technology, a multifunctional isocyanate is allowed to diffuse in the pores of a preformed alginate wet gel, where it reacts with the –OH groups on the surface of the alginate network and the water molecules adsorbed on it, yielding a polyurea/polyurethane-crosslinked alginate (X-alginate) aerogel [27,28,29,30]. Coating the alginate framework with polyurea makes X-alginate aerogels mechanically strong and extremely stable in water (including seawater). These properties have resulted in applications in environmental remediation, for example, by removing heavy metals, including lead and uranium, from various waters [20,31] and in biomedicine, for example, as medical implants [32].

Another approach for the modification of polysaccharides is by using their functional (e.g., –OH) groups as grafting sites for biocompatible polymers (e.g., polyesters). This technology has mainly been employed on cellulose, although it has been reported for other polysaccharides as well [33,34,35,36,37,38,39]. Polylactide (PLA) grafting on cellulose mainly increases its hydrophobicity [35] and produces amphiphilic materials suitable for drug delivery [36] or for carrying essential oil aromas [37]. PLA grafting on cellulose substrates can also produce materials with tunable thermal properties [38] or increase cellulose compatibility with polymer matrices in composite materials [39]. When it comes to alginate, although there are examples of alginate/PLA composite materials [40,41,42,43], no graft copolymers have been reported.

With an eye on new biocompatible aerogels, here we explore a strategy for the modification of the alginate network by reacting *DL*-lactide (LA) with pre-formed M-alginate (M = Ca^2+^, Co^2+^, Ni^2+^, Cu^2+^) gels towards the synthesis of polylactide- (PLA-) grafted materials. The selection of the gelation metal ions was made on the basis of their potential biomedical and/or environmental applications.

## 2. Materials and Methods

Two different sodium alginate sources were used. The two types of sodium alginate differed in their guluronate (G) content, which was determined with circular dichroism [44]. Sodium alginate purchased from ACROS (Geel, Belgium) had a 41% G content and will be referred to as G41, while sodium alginate purchased from Duchefa Biochemie (Haarlem, The Netherlands) had a 56% G content and will be referred to as G56. CaCl_2_·2H_2_O, CoCl_2_·6H_2_O, Ni(NO_3_)_2_·6H_2_O and CuCl_2_·2H_2_O were purchased from Sigma (Saint Louis, MO, USA). *DL*-lactide was purchased from Tokyo Chemical Industry (Tokyo, Japan), and it was recrystallized twice from acetone and dried under vacuum for 24 h prior to use. Stannous octoate (tin (II) 2-ethylhexanoate) was purchased from Alfa Aesar (Karlsruhe, Germany). MeCN (HPLC grade) was purchased from Fisher (Loughborough, UK), and it was distilled over CaH_2_ and degassed by three freeze–pump–thaw cycles prior to use. Acetone was purchased from Fisher (Loughborough, UK) and was used as received.

Drying was carried out in an autoclave (E3100, Quorum Technologies, East Sussex, UK). Wet gels were placed in the autoclave at 12 °C and were covered with acetone. Liquid CO_2_ was allowed in the autoclave; acetone was drained out as it was being displaced by liquid CO_2_ (5×; 1 per 30 min). Afterward, the temperature of the autoclave was raised to 45 °C and was maintained for 1 h. Finally, pressure was gradually released, allowing supercritical fluid (SCF) CO_2_ to escape as a gas, leaving dry gels (aerogels).

^13^C Cross-Polarization Magic Angle Spinning (CPMAS) NMR spectra were obtained with a 600 MHz Varian spectrometer (Varian, Palo Alto, CA, USA) operating at 150.80 MHz for ^13^C. For ^13^C ramped CPMAS spectra, the spinning rate used was 5 KHz, and the temperature was set at 25 °C. ATR-FTIR spectra were obtained with a Perkin Elmer Spectrum 100 Spectrometer.

Thermogravimetric analysis (TGA) was performed using a Q50 TGA model from TA instruments (TA Instruments-Waters LLC, New Castle, DE, USA). Samples were placed in platinum crucibles. An empty platinum crucible was used as a reference. Samples were heated from ambient temperatures to 800 °C in a 60 mL/min flow of N_2_ at a heating rate of 10 °C/min.

The glass transition temperatures were obtained by differential scanning calorimetry (DSC) using a 2910 modulated DSC model from TA instruments. The samples were heated or cooled at a rate of 10 °C/min from ~25 to 500 °C. The samples were annealed at 200 °C for 10 min and the second heating results were obtained in all cases.

N_2_-sorption and CO_2_-adsorption measurements were made on a Micromeritics Tristar II 3020 surface area and porosity analyzer (Micromeritics, Norcross, GA, USA). Skeletal densities (*ρ*_s_) were determined by He pycnometry, using a Micromeritics AccuPyc II 1340 pycnometer (Micromeritics, Norcross, GA, USA). Bulk densities (*ρ*_b_) of the samples were calculated from their weight and natural dimensions.

Scanning electron microscopy (SEM, JEOL Akishima, Tokyo, Japan) characterization was conducted with Pt-coated samples adhered onto a conductive double-sided adhesive carbon tape, using a high resolution FESEM JEOL JSM 7401f.

### Synthesis of PLA-Grafted M-Alginate (g-M-Alginate) Aerogels

A solution of sodium alginate (G41 or G56) in H_2_O (2% *w*/*w*) was prepared by dissolving sodium alginate (2.00 g) in H_2_O (98.00 g) at 25 °C. The solution (5 mL; containing 102 mg of sodium alginate, 1.17 mmol of –OH groups) was added dropwise, using a 25-mL burette, to 20 mL of a 0.2 M solution of a metal salt (CaCl_2_·2H_2_O, CoCl_2_·6H_2_O, Ni(NO_3_)_2_·6H_2_O or CuCl_2_·2H_2_O) under mild magnetic stirring. Spherical M-alginate (M: Ca^2+^, Co^2+^, Ni^2+^, Cu^2+^) hydrogel beads were formed instantly and were left to age for 24 h. Afterward, they were stepwise solvent-exchanged with MeCN/H_2_O mixtures (30, 60, 90% *v*/*v*) and then with dry acetonitrile (4×). A given amount of *DL*-lactide (LA) (e.g., 3.36 g, 23.3 mmol for LA/–OH molar ratio equal to 20) was dissolved in 20 mL dry MeCN, 162 μL Sn(oct)_2_ were added and the mixture was degassed. M-alginate beads were immersed in the solution containing the monomer and the catalyst, left for 18 h for the solution to diffuse inside the beads and then the mixture was refluxed for 18 h under inert atmosphere. After the end of the reaction, the beads were solvent-exchanged with acetone (4×, 3 h each, the volume of the solvent was equal to 4× the volume of the beads) and were dried from SCF CO_2_ to provide the corresponding PLA-grafted M-alginate (referred to as g-M-alginate; g: PLA-grafted, M: Ca^2+^, Co^2+^, Ni^2+^, Cu^2+^) aerogels. The corresponding native M-alginate aerogels were also prepared for comparison purposes.

## 3. Results and Discussion

### 3.1. Preparation and Chemistry of PLA-Grafted M-Alginate (g-M-Alginate) Aerogels

g-M-alginate beads (M: Ca^2+^, Co^2+^, Ni^2+^, Cu^2+^) were studied for two different types of sodium alginate and gelled by the corresponding M^2+^ ions, which substitute Na^+^ ions almost quantitatively (the sodium residue has been found lower than 0.1% *w*/*w*) [29]. The two types of sodium alginate differed in the G/M ratio, which was calculated using circular dichroism data [44], and it was found to be equal to 0.69 (G41), and 1.27 (G56), respectively.

Ca-alginate (G41) beads were reacted with different amounts of *DL*-lactide (LA), corresponding to different LA/–OH molar ratios (i.e., 10, 20, 30, 40 and 100) in order to study PLA formation in the final aerogel materials as a function of the amount of LA initially used. Co-, Ni- and Cu-alginate beads were studied by setting the LA/–OH molar ratio equal to 20, and in a few cases equal to 40. As it will be discussed below, the addition of higher amounts of LA does not change the chemical composition, or the material properties of the resulting grafted aerogels. The samples are referred to as g-M-alg-YY (GXX), where XX refers to the percent G content (41 or 56) and YY refers to the LA/–OH molar ratio. Grafting was not studied for Ni-alginate (G41) because the corresponding wet-gels suffered severe shrinkage in acetonitrile, which was the solvent for the reaction. This observation comes in agreement with our previous work [29] in which high shrinkage of low-G Ni-alginate was also observed.

**Scheme 1:**
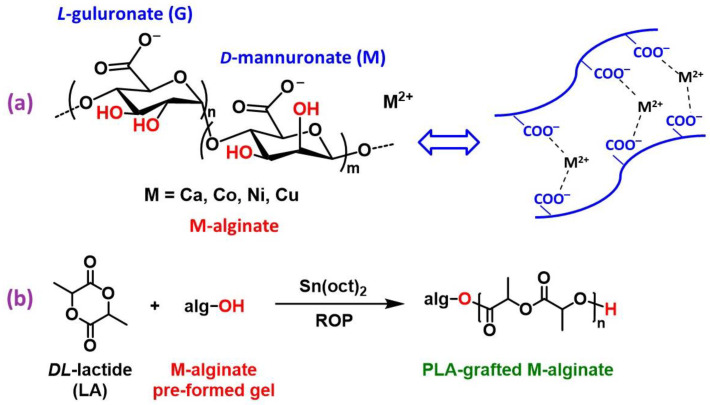
(**a**) Chemical structure of M-alginates (M: Ca^2+^, Co^2+^, Ni^2+^, Cu^2+^) and general scheme showing the gelation induced by M^2+^ ions (potential interactions with groups other than the carboxylates have not been included). (**b**) The reaction of grafting M-alginate gels with polylactide (PLA).

**Figure 1:**
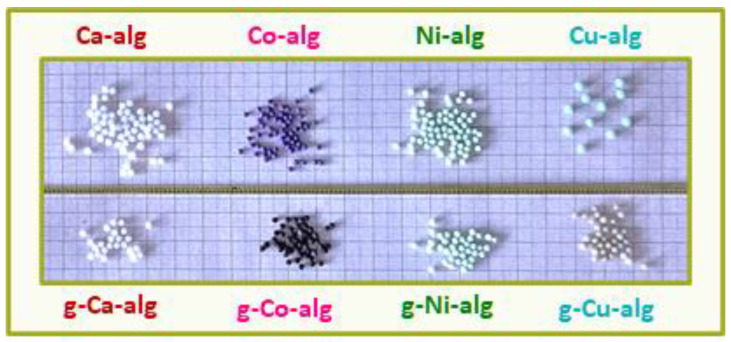
Optical photographs of M-alginate and g-M-alginate aerogels, as indicated.

Grafting of M-alginate gels with PLA was achieved via ring opening polymerization (ROP) of LA with stannous octoate (Sn(oct)_2_) as the catalyst by employing “grafting from” procedures [45,46,47]. The –OH groups of the alginate polymer chain were used as ROP initiators, as shown in Scheme 1. *DL*-lactide was used instead of the other lactide isomers, since an amorphous polymer is obtained in this case with a glass transition temperature close to 57 °C for high molecular weight materials [48]. The grafting reaction took place under reflux for 18 h. Optical photographs of the samples are shown in Figure 1 and size distributions on Figures S1–S4.

### 3.2. Chemical Characterization of PLA-Grafted M-Alginate (g-M-Alginate) Aerogels

**Figure 2:**
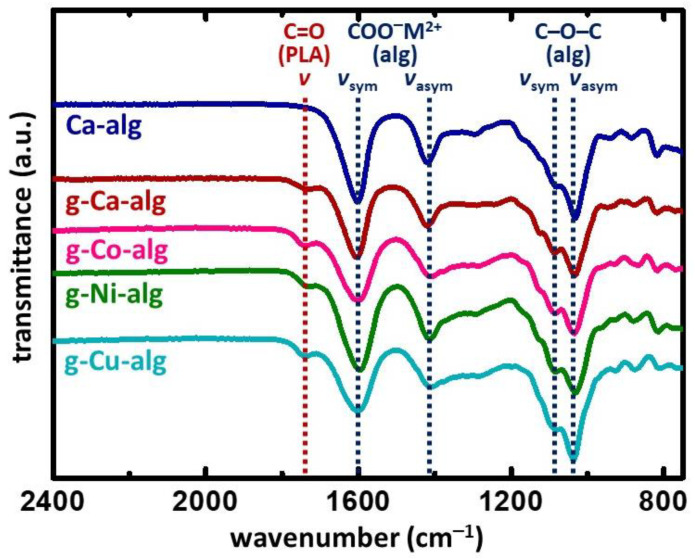
ATR-FTIR spectra of M-alginate and g-M-alginate aerogels, as indicated. Dotted lines mark the bands corresponding to the characteristic vibrations discussed in the text. The PLA content in the aerogels was in the range of 14–29% *w*/*w* (see Table 1).

ATR-FTIR spectra of both M-alginate and g-M-alginate aerogels show the characteristic vibrations of M-alginates (Figure 2). Specifically, the symmetric and asymmetric stretching vibrations of the carboxylate groups coordinated to M^2+^ ions appear at 1600 cm^−1^ and 1420 cm^−1^, respectively, while the corresponding stretching vibrations of the sugar ring C–O–C groups appear at 1080 cm^−1^ and 1030 cm^−1^. Additionally, g-M-alginate aerogels show a characteristic peak at 1734 cm^−1^, which corresponds to the C=O stretching of the PLA carbonyl groups [49].

**Figure 3:**
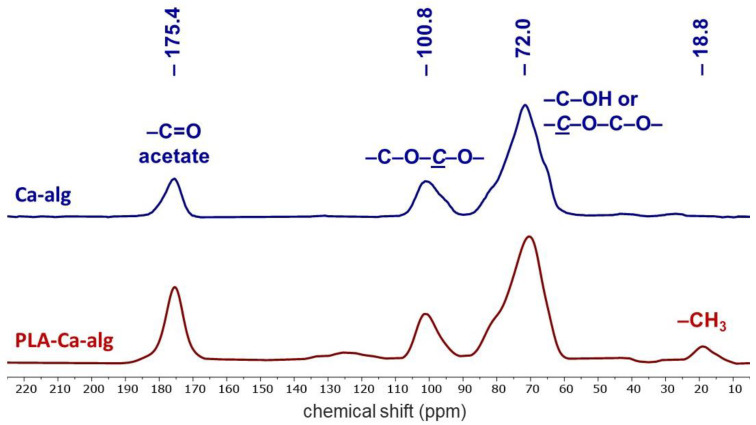
^13^C CPMAS NMR spectra of Ca-alginate and g-Ca-alginate aerogels, as indicated, with annotated characteristic peaks.

The ^13^C CPMAS NMR spectra of Ca-alginate and g-Ca-alginate aerogels (Figure 3) also show all the characteristic peaks expected for Ca-alginate [28,29]. The peak of the acetate carbonyls appears at 175.4 ppm. The peaks of the acetal carbons –C–O–*C*–O– on the alginate rings appear at 100.8 ppm, while the peaks corresponding to the alginate ring carbons attached to oxygen (–OH or ether) appear at 72.0 ppm, in agreement with previous observations [28,29]. In the spectrum of g-Ca-alginate, the ratio of the carbonyl peak to the two peaks of the ring carbons was larger compared to the same ratio in the spectrum of Ca-alginate alone. This finding and a new peak at 18.8 ppm, which is attributed to the methyl carbons of PLA [50], confirm the presence of PLA in the material.

The PLA content of g-M-alginate aerogels (Table 1) was calculated from their skeletal densities and those of the corresponding native M-alginate aerogels (reported in Table 2). For either g-Ca-alginate (G41) or (G56) aerogels that were prepared using different amounts of LA, corresponding to different LA/–OH molar ratios (i.e., 10, 20, 30, 40 and 100), the amount of PLA in the corresponding aerogels was very similar, in the range of 12 to 18% *w*/*w*, and it was not dependent on the LA/–OH molar ratio.

Regarding the other metals, it seems that not only the LA/–OH molar ratio but also the metal and the nature of the alginate are important for the PLA formation on the material (Table 1). Molar ratios higher than 40 were not studied in order to avoid the complication of transesterification side reactions [51,52,53]. In the case of g-Ni-alginate aerogels, the maximum PLA content was 14% *w*/*w*. In the cases of g-Co-alginate and g-Cu-alginate aerogels, better results were obtained with the G41 alginate and the maximum PLA content was 29% *w*/*w*, the highest among all materials prepared in this study. It is expected that, due to steric constraints, the polymerization of LA on the solid pre-formed alginate network is not kinetically favored. Another reason for the rather low grafting efficiency is transesterification side reactions, which result in soluble PLA chains. These chains are readily removed from the solution.

**Figure 4:**
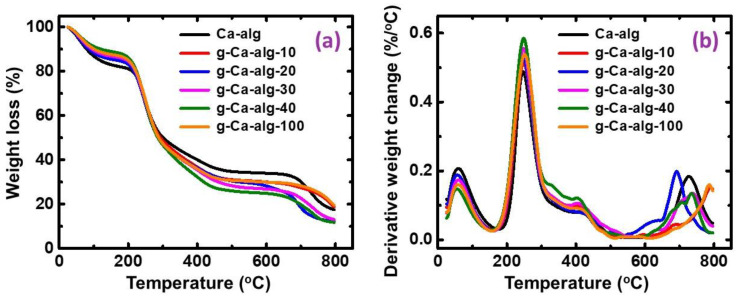
TGA (**a**) and DTG (**b**) curves for Ca-alginate and g-Ca-alginate (G41) aerogels with different LA/–OH molar ratios, as indicated.

The thermal stability of all aerogels was studied with thermogravimetric analysis (TGA) and differential thermogravimetry (DTG). For Ca-containing samples (Figure 4), all decomposition curves were similar. The samples exhibited a weight loss of 9–14% up to 100 °C, attributed to the moisture of the samples, and a total loss of 15–20% up to 200 °C. The main decomposition process started after 200 °C and consisted of three steps (as shown also from the DTG curves). At 800 °C, the residue was between 12 and 19% for all samples and corresponds to the residue of the alginate component. DTG showed the main decomposition peak at approximately 250 °C and a smaller and much broader peak at approximately 400 °C for all samples, while the third decomposition peak was between 692 and 786 °C. The results show the small contribution of PLA to the thermal decomposition of g-M-alginate aerogels. However, since PLA is known to be a relatively thermally sensitive polymer, the presence of the PLA chains is expected to slightly reduce the thermal stability of the aerogels. The mechanism of thermal decomposition of PLA is rather complex, initially involving the random chain scission to smaller chains, without leading to appreciable weight loss, followed by the main decomposition effect due to zipper-like depolymerization reactions and intermolecular and intramolecular transesterification reactions resulting in the formation of monomer and oligomers [54,55]. The decomposition process is greatly affected by remaining amounts of catalyst, metal ions and the nature of the polymer end groups [56,57]. In any case, the main thermal degradation takes place at the temperature range of 200–325 °C, which is similar to the range of decomposition of the alginates [58].

**Figure 5:**
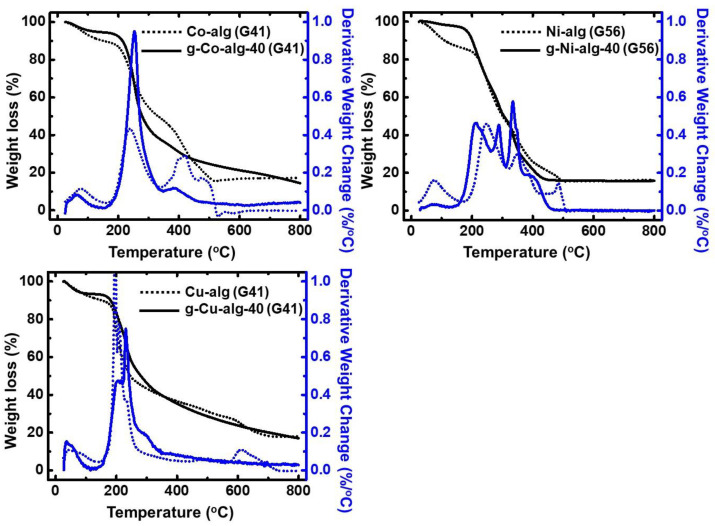
TGA (black lines) and DTG (blue lines) curves of M-alginate and g-M-alginate aerogels, as indicated.

For alginate aerogels gelled with Co, Ni and Cu (Figure 5), TGA and DTG data were in agreement with the literature [29]. The decomposition curves for each pair of M-alginate/g-M-alginate had only small differences, reflecting the small contribution of the PLA component to the thermal properties of g-M-alginate aerogels, as was discussed above.

The thermal properties of all Ca-containing samples were also studied with differential scanning calorimetry (DSC; Figure S5). Similar DSC traces were obtained for all grafted samples and the original Ca-alginate aerogel, which is in agreement with the results coming from the TGA analysis. This result was rather expected, since it has been shown for similar materials (i.e., PLA-grafted cellulose) that *T*_g_ depends on the PLA content [38], and for all g-Ca-alginate aerogels the PLA content is almost the same (Table 1). The *T*_g_ of PLA is expected to be at 57 °C for high molecular weight samples. This transition is very weak and can be hardly observed for samples g-Ca-alg-40 and g-Ca-alg-100. This is due to the rather low PLA content of the aerogels and the evaporation of humidity of the samples, which is completed at temperatures slightly higher than 100 °C The main decomposition event is then observed starting around 200 °C, as also shown by TGA.

### 3.3. Selected Material Properties of PLA-Grafted M-Alginate (g-M-Alginate) Aerogels

Material properties of all aerogels are summarized in Table 2. As has been discussed before, in order to study PLA formation in the final aerogel materials as a function of the amount of LA initially used, Ca-alginate (G41) beads were reacted with different amounts of LA, corresponding to different LA/–OH molar ratios (i.e., 10, 20, 30, 40 and 100). All resulting grafted materials had very similar properties, as expected from their very similar chemical composition (Table 1); they had low bulk densities (0.10–0.12 g cm^−3^) and high porosities (93–94% *v*/*v*), and they were all mostly macroporous materials (*V*_Total_ > *V*_1.7–300 nm_). Compared to the parent Ca-alginate aerogels, they have a bit higher bulk density and lower porosity, and no or little microporosity. Analogous observations can be made for g-Ca-alginate (G56) aerogels. These trends have been also observed in our previous studies for the crosslinking of M-alginates with polyurea [27,28].

For the other M-alginate materials, the LA/–OH molar ratios of 20 and 40 were studied. All resulting grafted aerogels had low bulk densities (0.08–0.24 g cm^−3^) and high porosities (86–96% *v*/*v*), and they were all mostly macroporous materials (*V*_Total_ > *V*_1.7–300 nm_), with some mesoporosity and little or no microporosity. In general, BET surface areas and average pore sizes did not change significantly after grafting. As also described above, grafting with PLA has little effect on the material properties of the g-M-alginate aerogels, which properties are mostly determined by the corresponding parent M-alginate aerogels.

In all cases, N_2_-sorption isotherms (Figures S6–S9) showed narrow hysteresis loops and did not reach saturation, as expected for macroporous materials with only a small amount of mesoporosity. The Barrett–Joyner–Halenda (BJH) curves (Figures S6–S9, insets), for pores in the range of 1.7–300 nm, were quite broad and showed maxima at 33–34 nm for all materials, except for Cu-alginate (G57) (maximum at 27 nm).

The fact that grafting does not affect the material properties significantly is attributed not only to the low content of PLA but also to the fact that the PLA chains (linear polymer) that have grown on the M-alginate framework extend in the pores of g-M-alginates, but they do not coat the solid framework, as is the case with polyurea-crosslinked M-alginate aerogels [27,28,29,30]. The same has been observed previously with poly(methyl methacrylate)-grafted polydicyclopentadiene aerogels [59,60].

**Figure 6:**
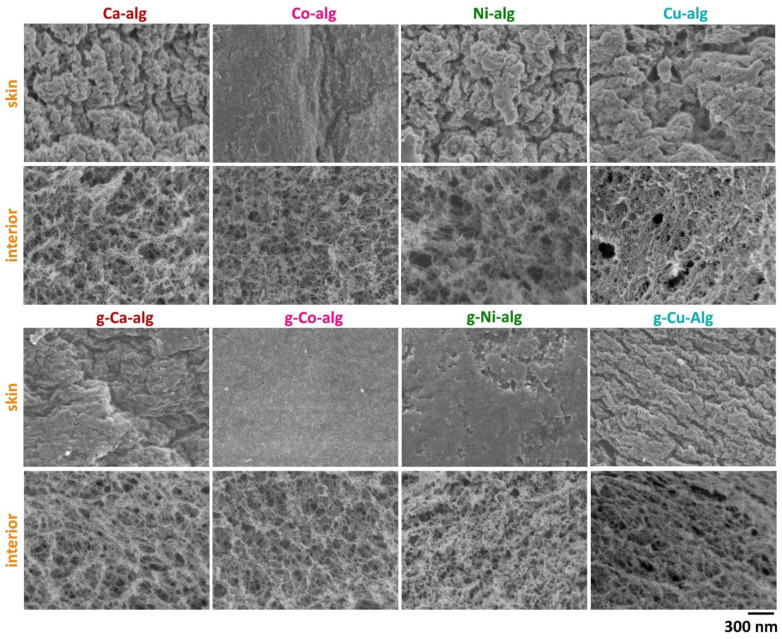
Representative SEM images of M-alginate and g-M-alginate aerogels, as indicated.

Representative SEM images (Figure 6) showed that all samples were fibrous, with denser structures on the surface of the beads than in their interior, in agreement with previous observations [28,29,61]. Grafting with PLA did not affect morphology, and M-alginate and g-M-alginate aerogels had very similar morphologies. This is a feature also observed with polyurea-crosslinked alginate aerogels [27,28,29]; in all cases, the morphology was determined primarily by the M-alginate framework. SEM images also confirm the presence of macropores in both M-alginate and g-M-alginate aerogels, which is in agreement with the N_2_-sorption data (Table 2).

## 4. Conclusions

The synthesis and material properties of PLA-grafted M-alginate (g-M-alginate; M: Ca^2+^, Co^2+^, Ni^2+^, Cu^2+^) aerogels are presented in this work. *DL*-lactide (LA) was attached on the surface of pre-formed M-alginate beads and was polymerized, using stannous octoate as the catalyst and the –OH groups of the alginate backbone as initiators/points of attachment. g-M-alginate aerogels are lightweight (bulk densities in the range 0.08–0.24 g cm^−3^), macroporous/mesoporous materials with high porosities (86–96% *v*/*v*) and BET surface areas in the range 154–542 m^2^ g^−1^, depending on the metal, the nature of the alginate framework and the PLA content. The latter was found at about 15% *w*/*w* for Ca- and Ni-based materials, and around 29% *w*/*w* for Co- and Cu-based materials. The morphology was fibrous in all cases. The major thermal degradation of g-M-alginate aerogels took place in the range of 200–400 °C, where both components (PLA and alginate) decompose, and the PLA chains had a small effect on the decomposition process of grafted versus native aerogels. In general, the material properties of g-M-alginate aerogels were not affected much by grafting, but they remained similar to those of the M-alginate component. This is attributed to the fact that the PLA chains consist of a linear polymer that does not keep on accumulating on itself and does not bridge its points of attachment on the g-M-alginate framework as in the case of polyurea-crosslinked M-alginate aerogels; instead, PLA forms a monolayer on the skeletal framework, the most significant effect of which is to block access to the micropores (notice that both the skeletal density and the micropore surface area decrease). Overall, this work has demonstrated a new methodology for the functionalization of alginate aerogels that opens the way to the synthesis of polylactide-crosslinked alginate aerogels with the use of multifunctional monomers.

## Figures and Tables

**Table 1:** PLA content of g-M-alginate aerogels.

Sample	PLA Content (%)
g-Ca-alg-10 (G41)	12
g-Ca-alg-20 (G41)	17
g-Ca-alg-30 (G41)	13
g-Ca-alg-40 (G41)	18
g-Ca-alg-100 (G41)	17
g-Ca-alg-20 (G56)	13
g-Ca-alg-40 (G56)	14
g-Co-alg-20 (G41)	2
g-Co-alg-40 (G41)	29
g-Co-alg-20 (G56)	2
g-Ni-alg-20 (G56)	2
g-Ni-alg-40 (G56)	14
g-Cu-alg-20 (G41)	15
g-Cu-alg-40 (G41)	29
g-Cu-alg-20 (G56)	6

PLA content calculated according to formula: %PLA (*w*/*w*) = [(*ρ*_s PLA_/*ρ*_s g-M-alg_) × (*ρ*_s M-alg_ − *ρ*_s g-M-alg_)/(*ρ*_s M-alg_ − *ρ*_s PLA_)] × 100 [27,28,29]. Values for *ρ*_s M-alg_ and *ρ*_s g-M-alg_ were taken from Table 2, and the value for *ρ*_s PLA_ was measured equal to 1.26 g cm^−3^.

**Table 2:** Selected material properties of M-alginate and g-M-alginate aerogel beads.

Sample ^a^	Bulk Density *ρ*_b_ (g cm^−3^)	Skeletal Density *ρ*_s_ (g cm^−3^)	Porosity ^b^ *Π* (% *v*/*v*)	BET Surf. Area *σ* (m^2^ g^−1^) (Micropore Surf. Area) ^c^	*V*_Total_ ^d^ (*V*_1.7–300nm_) ^e^ (cm^3^ g^−1^)	Av. Pore Diam. ^f^ (4*V*_Total_/*σ*) (nm)
Ca-alg (G41)	0.076 ± 0.006	1.97 ± 0.03	96	340 (53)	13 (2.0)	23 (149)
g-Ca-alg-10 (G41)	0.12 ± 0.01	1.85 ± 0.02	94	377 (8)	8.1 (2.0)	21 (86)
g-Ca-alg-20 (G41)	0.104 ± 0.006	1.80 ± 0.02	94	433	9.1 (1.2)	12 (84)
g-Ca-alg-30 (G41)	0.10 ± 0.01	1.84 ± 0.02	94	425	9.2 (1.2)	13 (86)
g-Ca-alg-40 (G41)	0.11 ± 0.01	1.79 ± 0.02	94	390	8.5 (1.5)	16 (88)
g-Ca-alg-100 (G41)	0.12 ± 0.01	1.80 ± 0.01	93	398	7.8 (1.5)	16 (78)
Ca-alg (G56)	0.068 ± 0.006	1.95 ± 0.02	97	472 (88)	14 (1.6)	15 (120)
g-Ca-alg-20 (G56)	0.08 ± 0.02	1.82 ± 0.02	96	438 (17)	12 (1.4)	14 (109)
g-Ca-alg-40 (G56)	0.061 ± 0.005	1.81 ± 0.01	97	422 (12)	16 (1.1)	11 (150)
Co-alg (G41)	0.18 ± 0.02	1.93 ± 0.01	91	286 (49)	5.0 (2.1)	29 (70)
g-Co-alg-20 (G41)	0.18 ± 0.01	1.91 ± 0.02	91	250 (18)	5.0 (1.4)	23 (80)
g-Co-alg-40 (G41)	0.24 ± 0.04	1.67 ± 0.01	86	160	3.6 (1.3)	32 (89)
Co-alg (G56)	0.14 ± 0.01	2.05 ± 0.02	93	289 (54)	6.6 (1.1)	15 (92)
g-Co-alg-20 (G56)	0.17 ± 0.03	2.04 ± 0.04	92	229	5.4 (0.6)	12 (94)
Ni-alg (G56)	0.14 ± 0.01	1.93 ± 0.01	93	275 (45)	6.6 (0.9)	15 (96)
g-Ni-alg-20 (G56)	0.12 ± 0.02	1.91 ± 0.02	94	254 (21)	7.8 (0.7)	12 (123)
g-Ni-alg-40 (G56)	0.12 ± 0.04	1.80 ± 0.01	93	154 (12)	7.8 (0.8)	22 (202)
Cu-alg (G41)	0.068 ± 0.006	2.06 ± 0.02	97	568 (84)	14 (3.7)	27 (100)
g-Cu-alg-20 (G41)	0.08 ± 0.01	1.88 ± 0.02	96	542 (63)	12 (3.8)	28 (88)
g-Cu-alg-40 (G41)	0.09 ± 0.02	1.74 ± 0.06	95	510 (19)	11 (3.2)	25 (83)
Cu-alg (G56)	0.056 ± 0.006	2.12 ± 0.06	97	584 (109)	17 (2.6)	19 (119)
g-Cu-alg-20 (G56)	0.09 ± 0.01	2.03 ± 0.02	96	494	11 (2.9)	24 (86)

^a^ The concentration of the sodium alginate solution was 2% *w*/*w*. The two different alginates used in this study are denoted in parentheses. ^b^ Porosity was calculated according to formula (*ρ*_s_ − *ρ*_b_)/*ρ*_s_, where *ρ*_s_ is the skeletal density and *ρ*_b_ is the bulk density. ^c^ Micropore surface area via *t*-plot analysis, according to the Harkins and Jura model. ^d^ Total pore volume calculated according to formula: 1/*ρ*_b_ − 1/*ρ*_s_. ^e^ Cumulative volume of pores between 1.7 and 300 nm from N_2_-sorption data and the BJH desorption method. ^f^ Calculated by the 4 *V*/*σ* method; *V* was set equal to the maximum volume of N_2_ adsorbed along the isotherm as *P*/*P*_o_ → 1.0. For the number in parentheses, *V* was set equal to *V*_Total_ from the previous column.

## Data Availability

The data presented in this study are available in the main body of the article and in Supplementary Materials. Additional data presented in this study are available upon request from the corresponding authors.

## Supplementary Materials

The following supporting information can be downloaded at: https://www.mdpi.com/article/10.3390/polym14061254/s1, Figure S1: Size distributions of Ca-alginate and g-Ca-alginate (G41) aerogels with different LA/–OH molar ratios, as indicated (diameters measured with ImageJ; histograms were calculated using OriginPro 9.0). Mean diameter and sample size (N) are shown on the Figure; Figure S2: Size distributions of Ca-alginate and g-Ca-alginate (G56) aerogels with different LA/–OH molar ratios, as indicated (diameters measured with ImageJ; histograms were calculated using OriginPro 9.0). Mean diameter and sample size (N) are shown on the Figure; Figure S3: Size distributions of M-alginate and g-M-alginate (G41) aerogels with different LA/–OH molar ratios, as indicated (diameters measured with ImageJ; histograms were calculated using OriginPro 9.0). Mean diameter and sample size (N) are shown on the Figure; Figure S4: Size distributions of M-alginate and g-M-alginate (G56) aerogels with different LA/–OH molar ratios, as indicated (diameters measured with ImageJ; histograms were calculated using OriginPro 9.0). Mean diameter and sample size (N) are shown on the Figure; Figure S5: DSC thermograms for Ca-alginate and g-Ca-alginate (G41) aerogels with different LA/–OH molar ratios, as indicated; Figure S6: N_2_-sorption diagrams of Ca-alginate and g-Ca-alginate (G41) aerogels, as indicated. Insets show pore size distributions by the BJH method; Figure S7: N_2_-sorption diagrams of Ca-alginate and g-Ca-alginate (G56) aerogels, as indicated. Insets show pore size distributions by the BJH method; Figure S8: N_2_-sorption diagrams of M-alginate and g-M-alginate (G41) aerogels, as indicated. Insets show pore size distributions by the BJH method; Figure S9: N_2_-sorption diagrams of M-alginate and g-M-alginate (G56) aerogels, as indicated. Insets show pore size distributions by the BJH method.
