# Supplementary material for: Polylactide-Grafted Metal-Alginate Aerogels
Source: Polymers (Basel). 2022 Mar 21;14(6):1254. doi: 10.3390/polym14061254 (PMC8953683; doi:10.3390/polym14061254)
Supplement: Supplementary file 1 [file polymers-14-01254-s001.zip › polymers-1621360-supplementary.pdf]

polymers

MDPI

Article

Polylactide-Grafted Metal-Alginate Aerogels

Grigorios Raptopoulos [1,*], Ioannis Choinopoulos [2], Filippos Kontoes-Georgoudakis [2] and Patrina Paraskevopoulou [1,*]

1 Inorganic Chemistry Laboratory, Department of Chemistry, National and Kapodistrian University of Athens, Panepistimiopolis Zografou, 15771 Athens, Greece
2 Industrial Chemistry Laboratory, Department of Chemistry, National and Kapodistrian University of Athens, Panepistimiopolis Zografou, 15771 Athens, Greece; ichoinop@chem.uoa.gr (I.C.); fkontoes@chem.uoa.gr (F.K.-G.)
* Correspondence: grigorisrap@chem.uoa.gr (G.R.); paraskevopoulou@chem.uoa.gr (P.P.)

Citation: Raptopoulos, G.; Choinopoulos, I.; Kontoes-Georgoudakis, F.; Paraskevopoulou, P. Polylactide-Grafted Metal-Alginate Aerogels. *Polymers* **2022**, *14*, 1254. https://doi.org/10.3390/polym14061254

Academic Editor: Amir Ameli

Received: 16 February 2022
Accepted: 15 March 2022
Published: 21 March 2022

Publisher's Note: MDPI stays neutral with regard to jurisdictional claims in published maps and institutional affiliations.

Copyright: © 2022 by the authors. Submitted for possible open access publication under the terms and conditions of the Creative Commons Attribution (CC BY) license (https://creativecommons.org/licenses/by/4.0/).

Table of contents

	Page
**Figure S1.** Size distributions of Ca-alginate and g-Ca-alginate (G41) aerogels with different LA/–OH molar ratios, as indicated (diameters measured with ImageJ; histograms were calculated using OriginPro 9.0). Mean diameter and sample size (N) are shown on the Figure.	S2
**Figure S2.** Size distributions of Ca-alginate and g-Ca-alginate (G56) aerogels with different LA/–OH molar ratios, as indicated (diameters measured with ImageJ; histograms were calculated using OriginPro 9.0). Mean diameter and sample size (N) are shown on the Figure.	S3
**Figure S3.** Size distributions of M-alginate and g-M-alginate (G41) aerogels with different LA/–OH molar ratios, as indicated (diameters measured with ImageJ; histograms were calculated using OriginPro 9.0). Mean diameter and sample size (N) are shown on the Figure.	S4
**Figure S4.** Size distributions of M-alginate and g-M-alginate (G56) aerogels with different LA/–OH molar ratios, as indicated (diameters measured with ImageJ; histograms were calculated using OriginPro 9.0). Mean diameter and sample size (N) are shown on the Figure.	S5
**Figure S5.** DSC thermograms for Ca-alginate and g-Ca-alginate (G41) aerogels with different LA/–OH molar ratios, as indicated.	S6
**Figure S6.** N_2-sorption diagrams of Ca-alginate and g-Ca-alginate (G41) aerogels, as indicated. Insets show pore size distributions by the BJH method.	S8
**Figure S7.** N_2-sorption diagrams of Ca-alginate and g-Ca-alginate (G56) aerogels, as indicated. Insets show pore size distributions by the BJH method.	S9
**Figure S8.** N_2-sorption diagrams of M-alginate and g-M-alginate (G41) aerogels, as indicated. Insets show pore size distributions by the BJH method.	S10
**Figure S9.** N_2-sorption diagrams of M-alginate and g-M-alginate (G56) aerogels, as indicated. Insets show pore size distributions by the BJH method.	S11

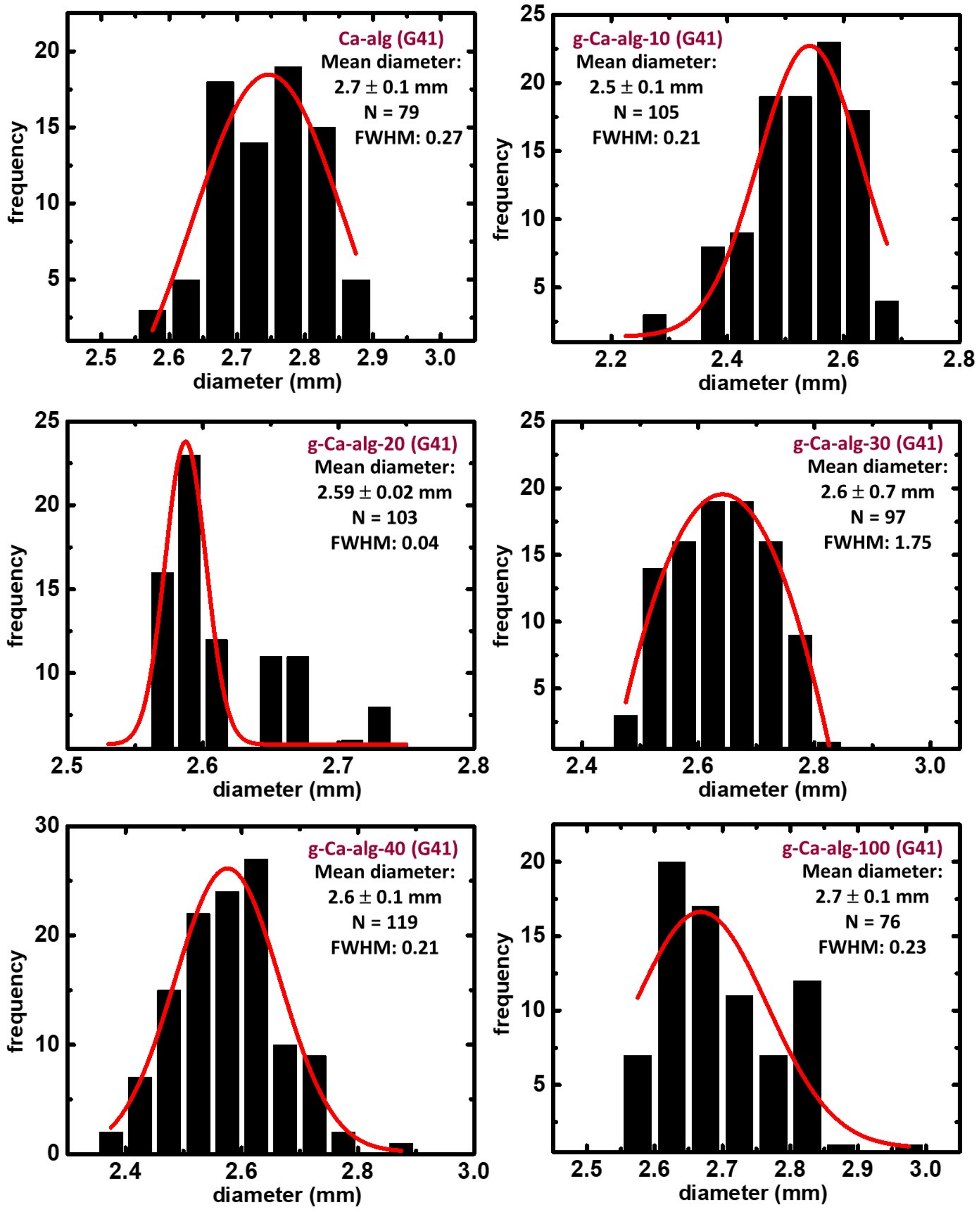

Figure S1. Size distributions of Ca-alginate and g-Ca-alginate (G41) aerogels with different LA/–OH molar ratios, as indicated (diameters measured with ImageJ; histograms were calculated using OriginPro 9.0). Mean diameter and sample size (N) are shown on the Figure.

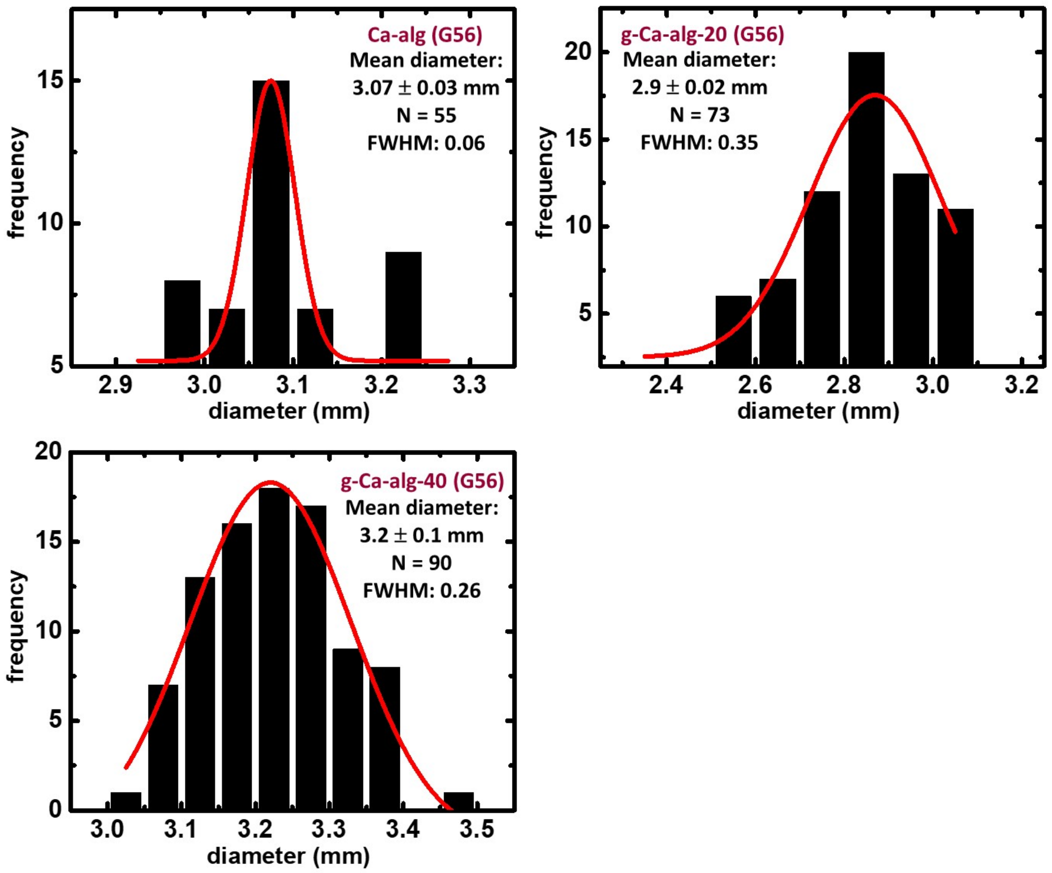

Figure S2. Size distributions of Ca-alginate and g-Ca-alginate (G56) aerogels with different LA/–OH molar ratios, as indicated (diameters measured with ImageJ; histograms were calculated using OriginPro 9.0). Mean diameter and sample size (N) are shown on the Figure.

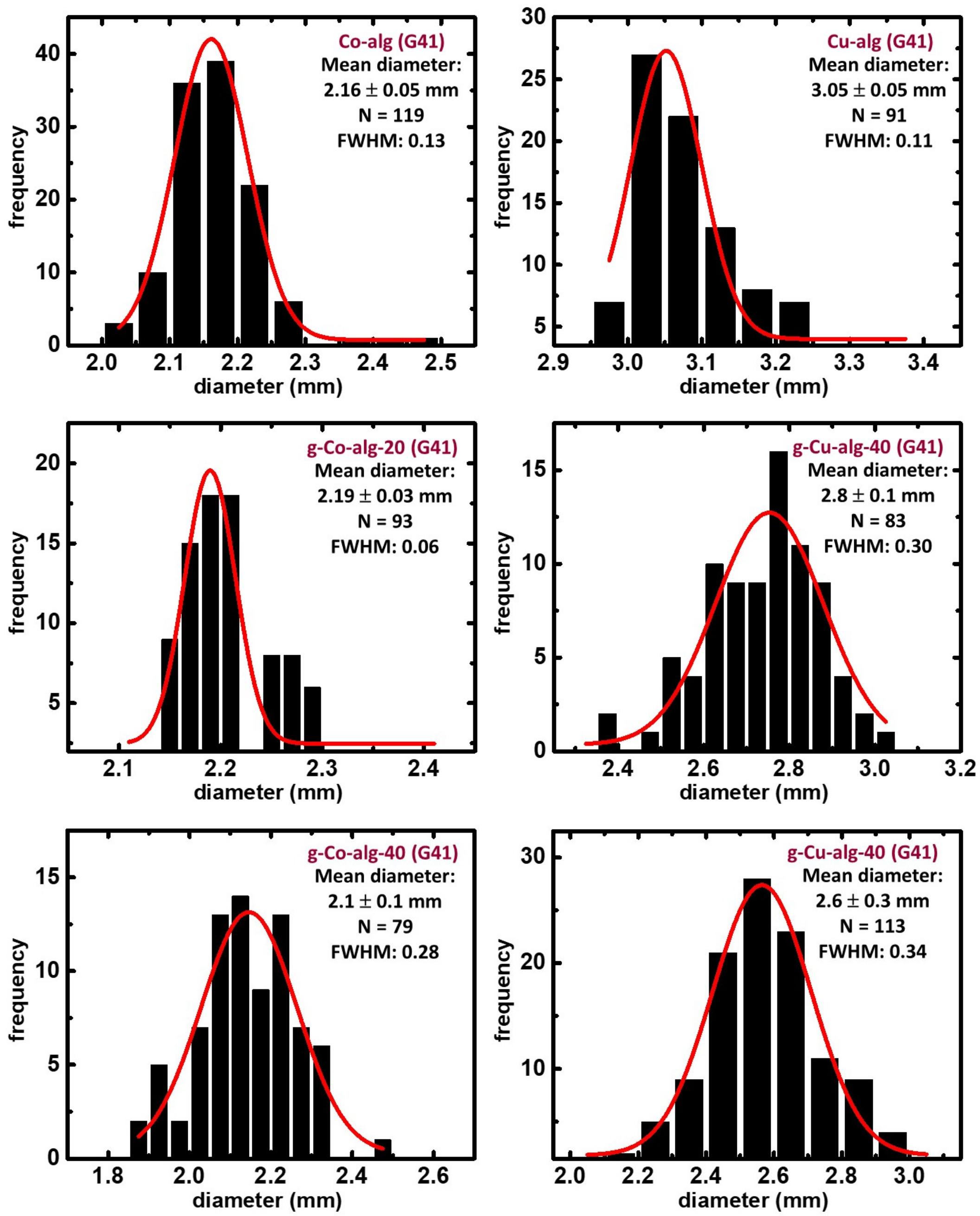

Figure S3. Size distributions of M-alginate and g-M-alginate (G41) aerogels with different LA/–OH molar ratios, as indicated (diameters measured with ImageJ; histograms were calculated using OriginPro 9.0). Mean diameter and sample size (N) are shown on the Figure.

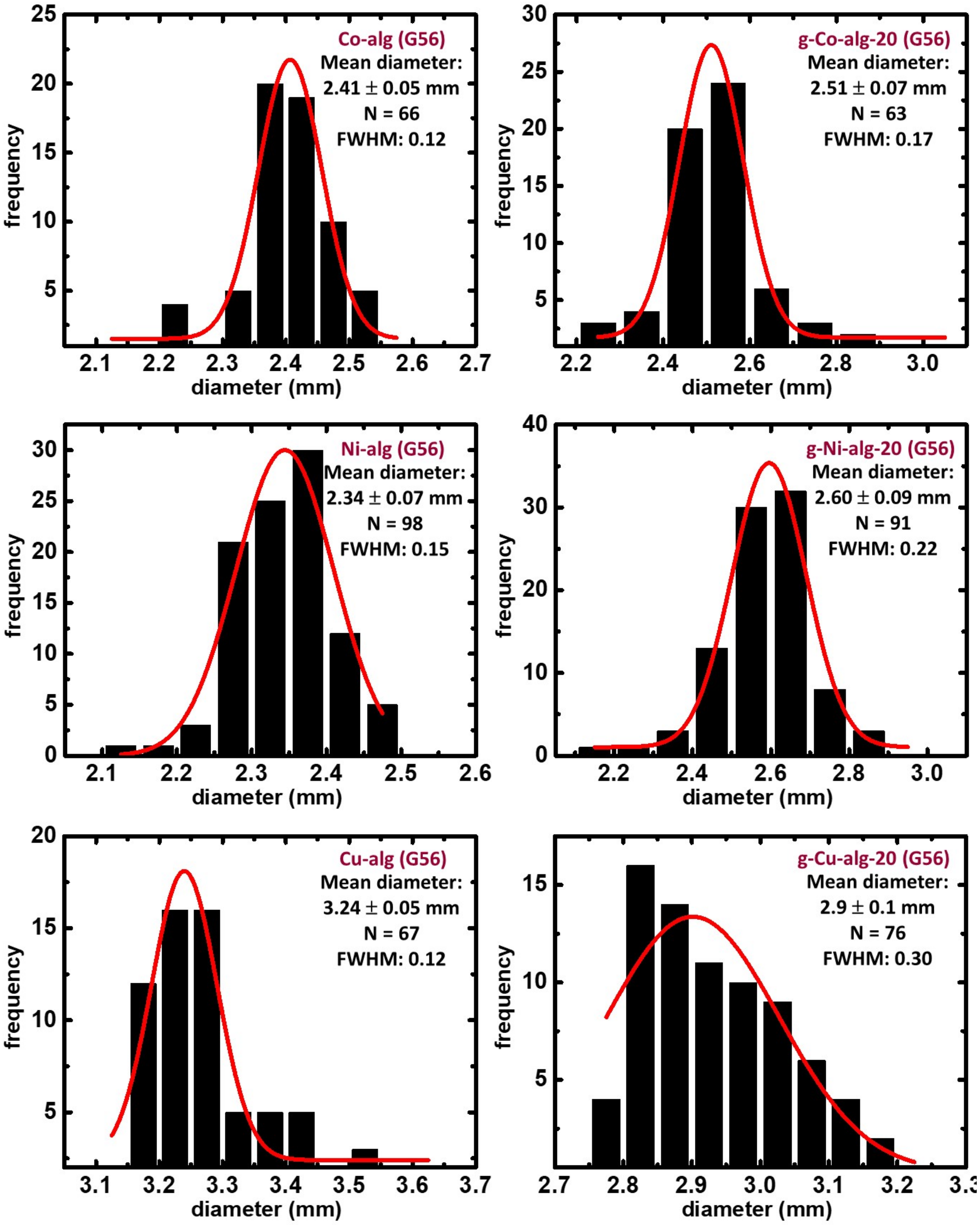

Figure S4. Size distributions of M-alginate and g-M-alginate (G56) aerogels with different LA/–OH molar ratios, as indicated (diameters measured with ImageJ; histograms were calculated using OriginPro 9.0). Mean diameter and sample size (N) are shown on the Figure.

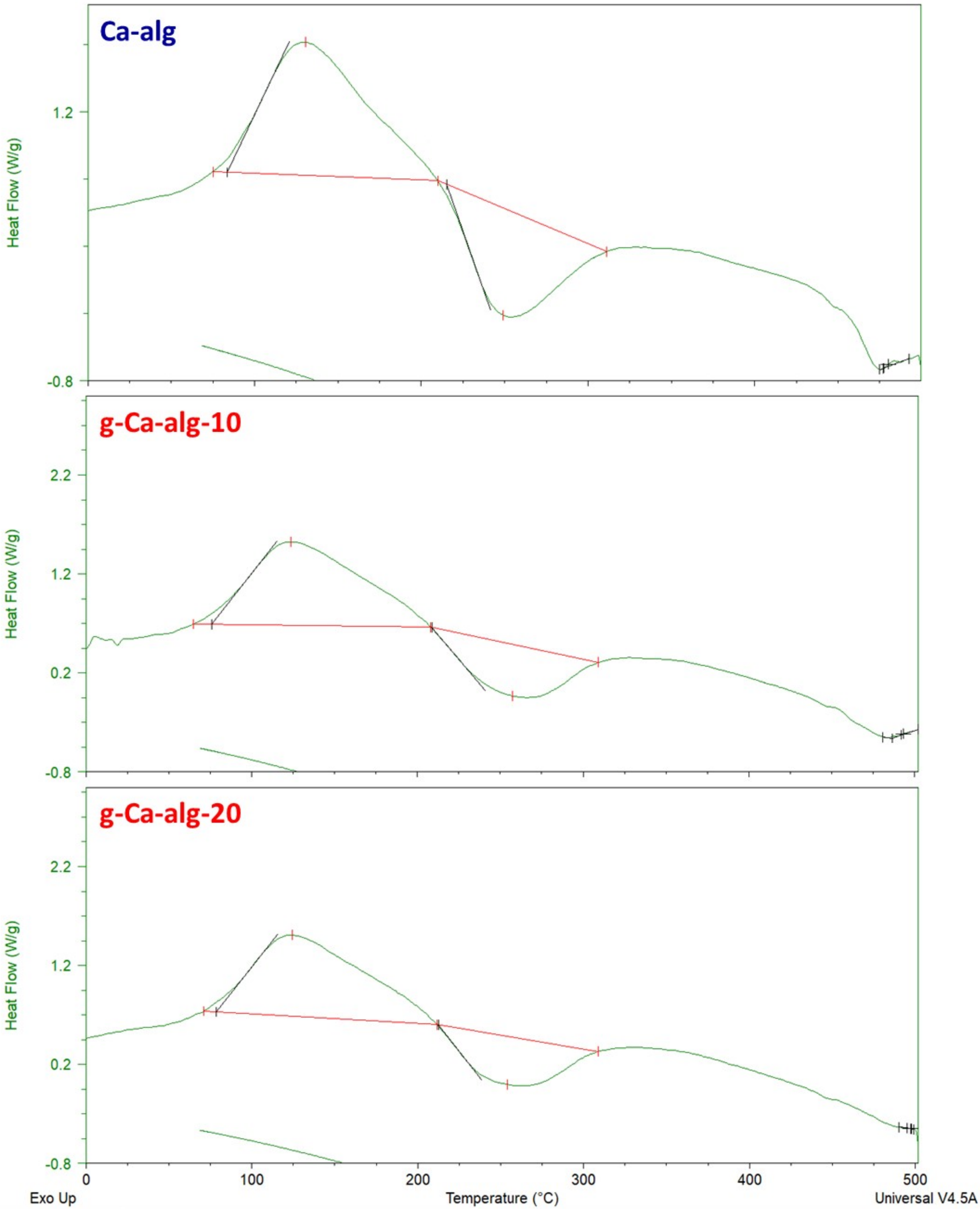

Figure S5. Cont.

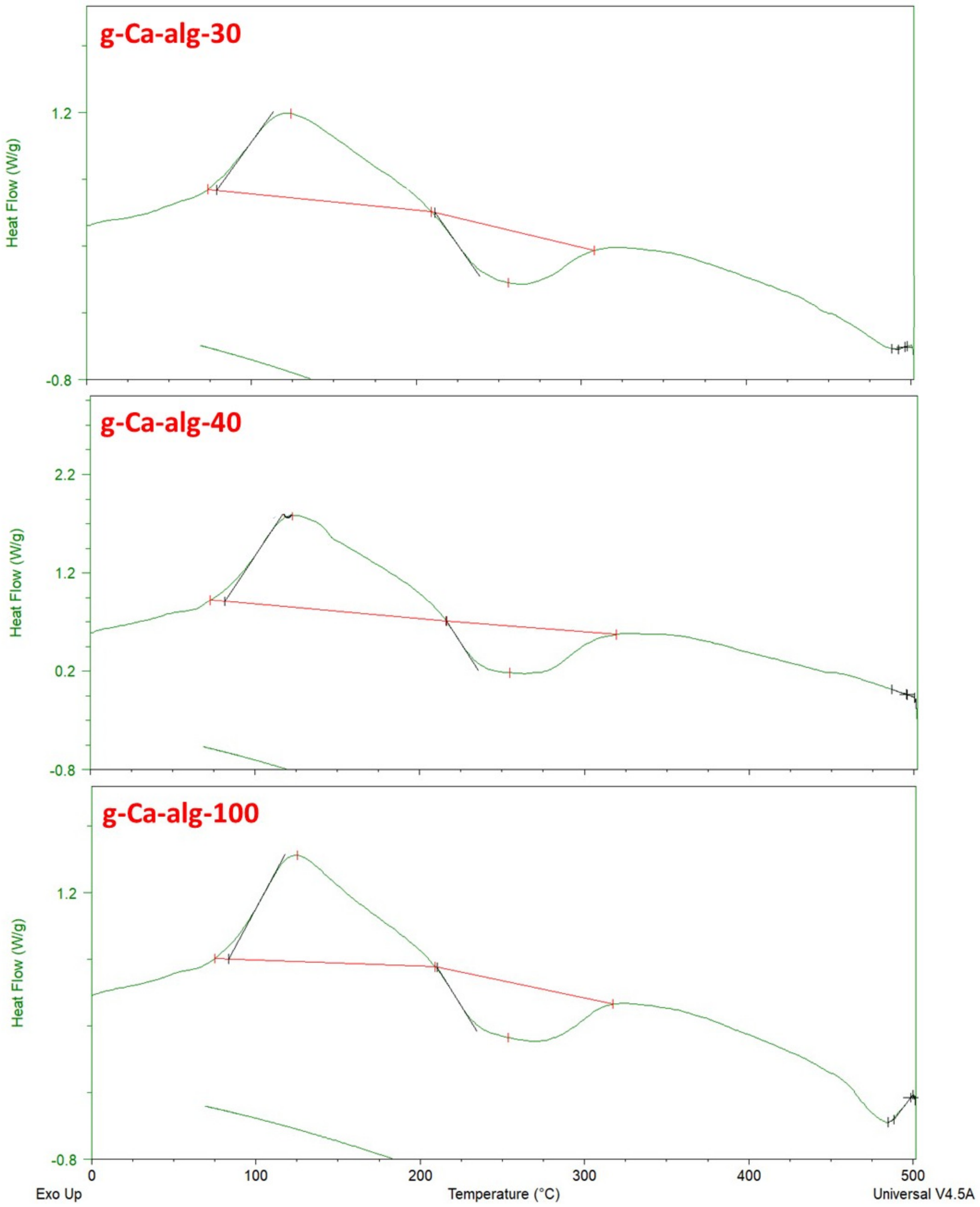

Figure S5. DSC thermograms for Ca-alginate and g-Ca-alginate (G41) aerogels with different LA/–OH molar ratios, as indicated.

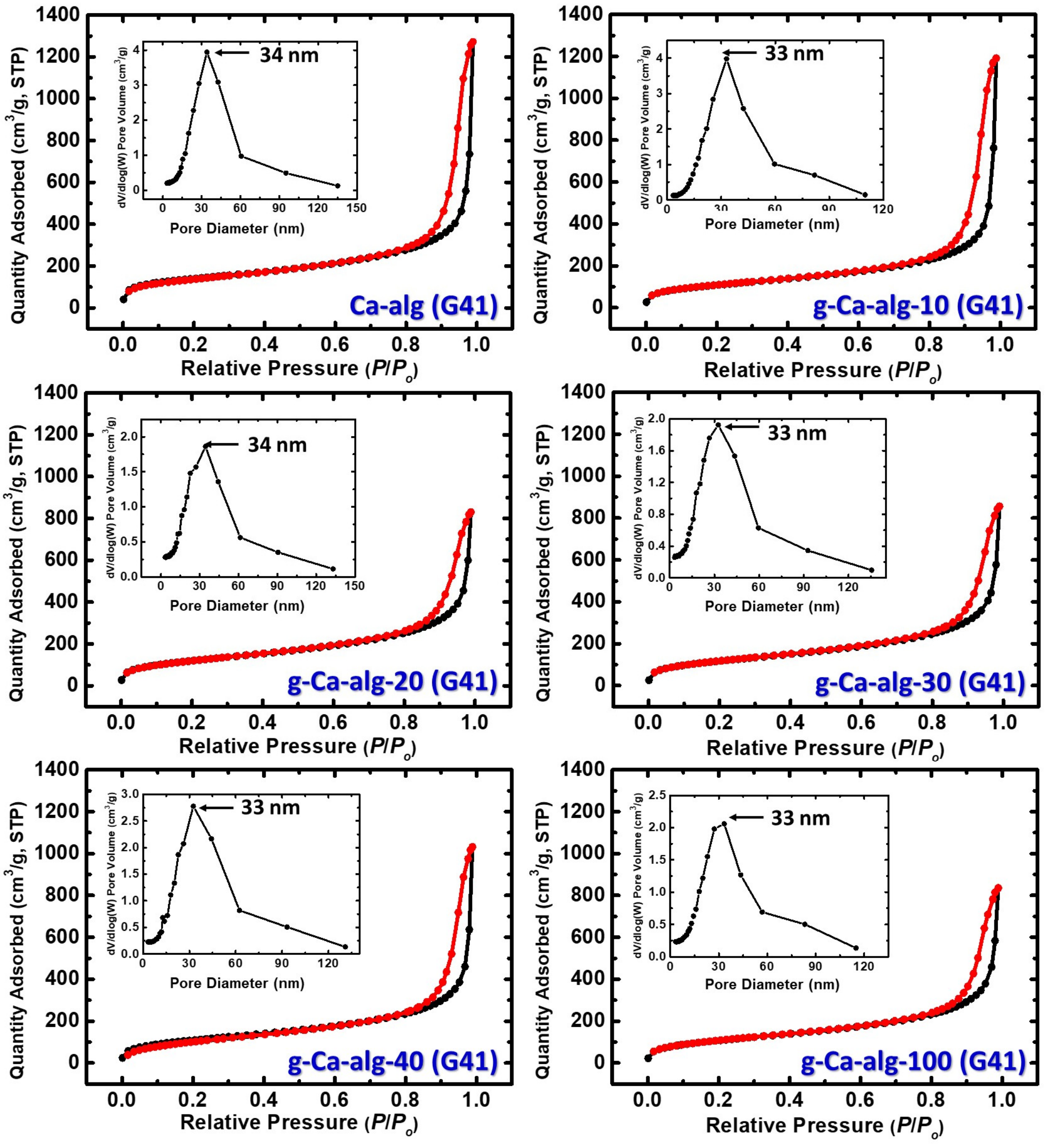

Figure S6. N_2-sorption diagrams of Ca-alginate and g-Ca-alginate (G41) aerogels, as indicated. Insets show pore size distributions by the BJH method.

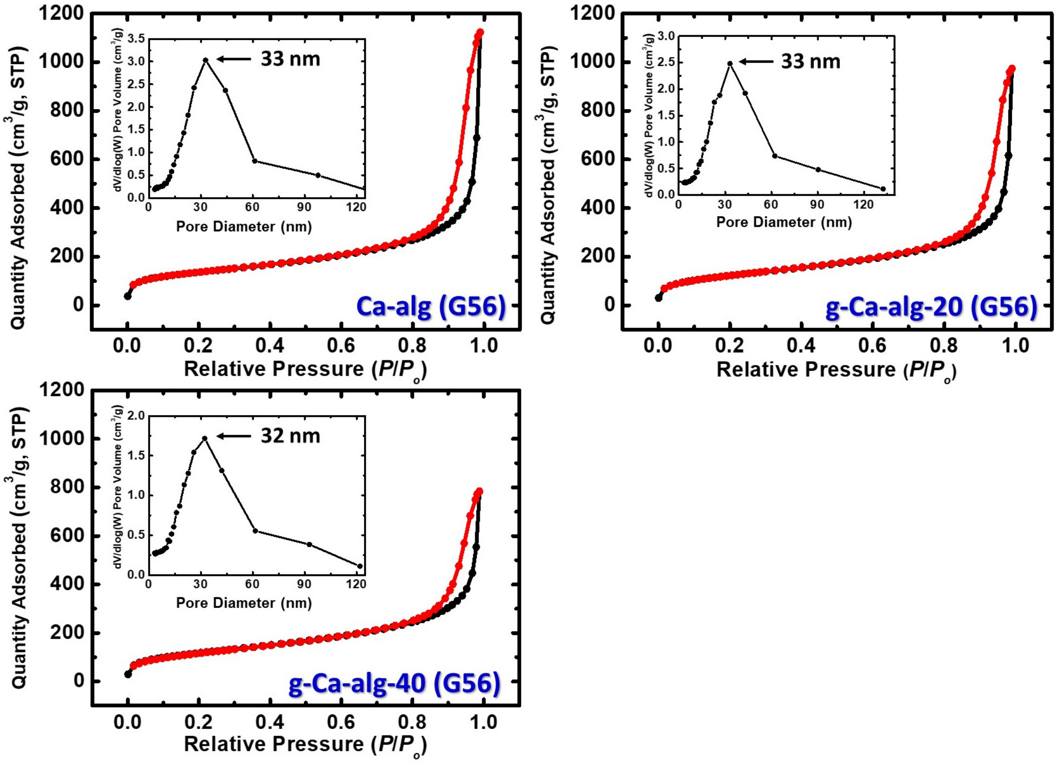

Figure S7. N_2-sorption diagrams of Ca-alginate and g-Ca-alginate (G56) aerogels, as indicated. Insets show pore size distributions by the BJH method.

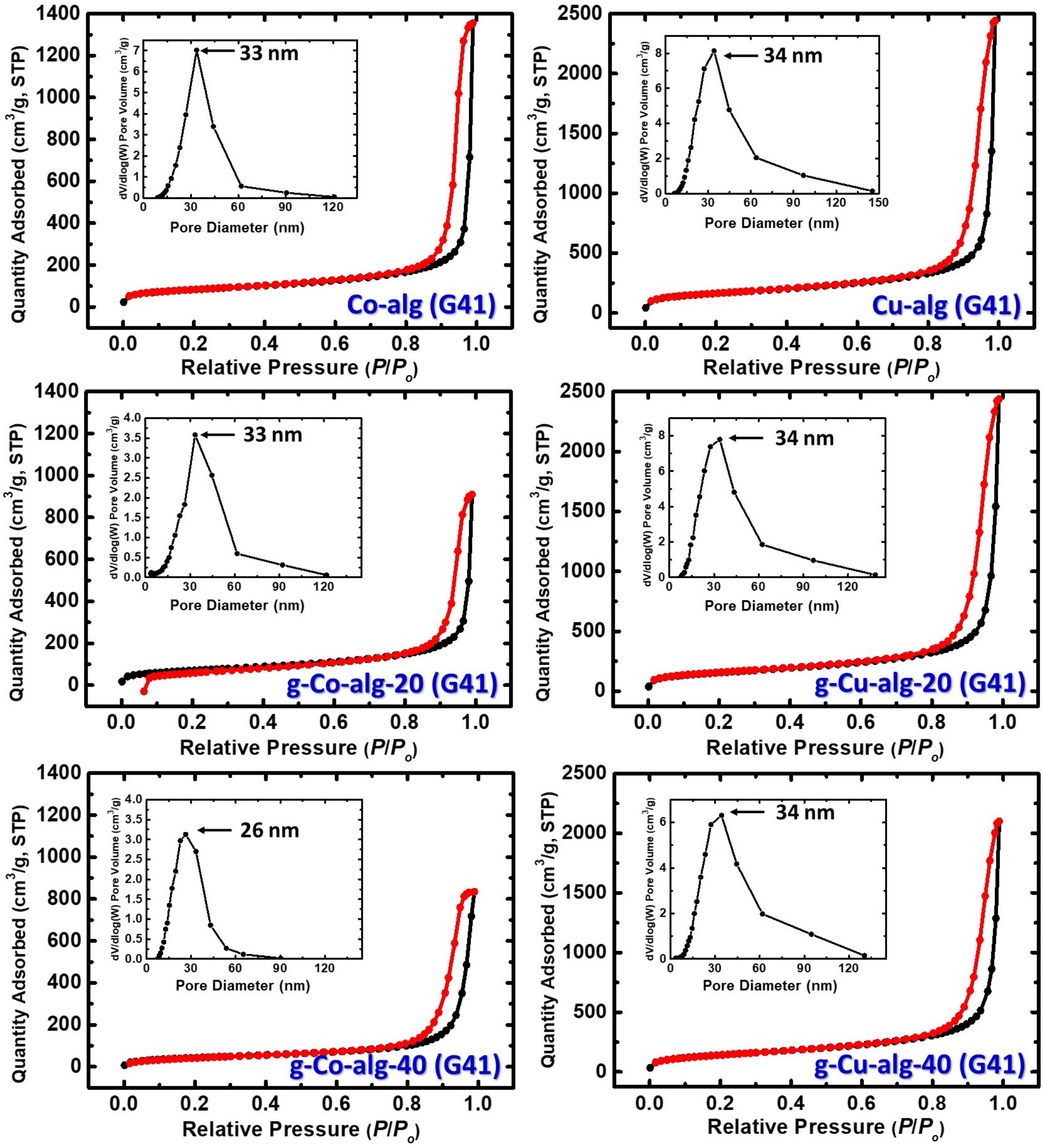

Figure S8. N_2-sorption diagrams of M-alginate and g-M-alginate (G41) aerogels, as indicated. Insets show pore size distributions by the BJH method.

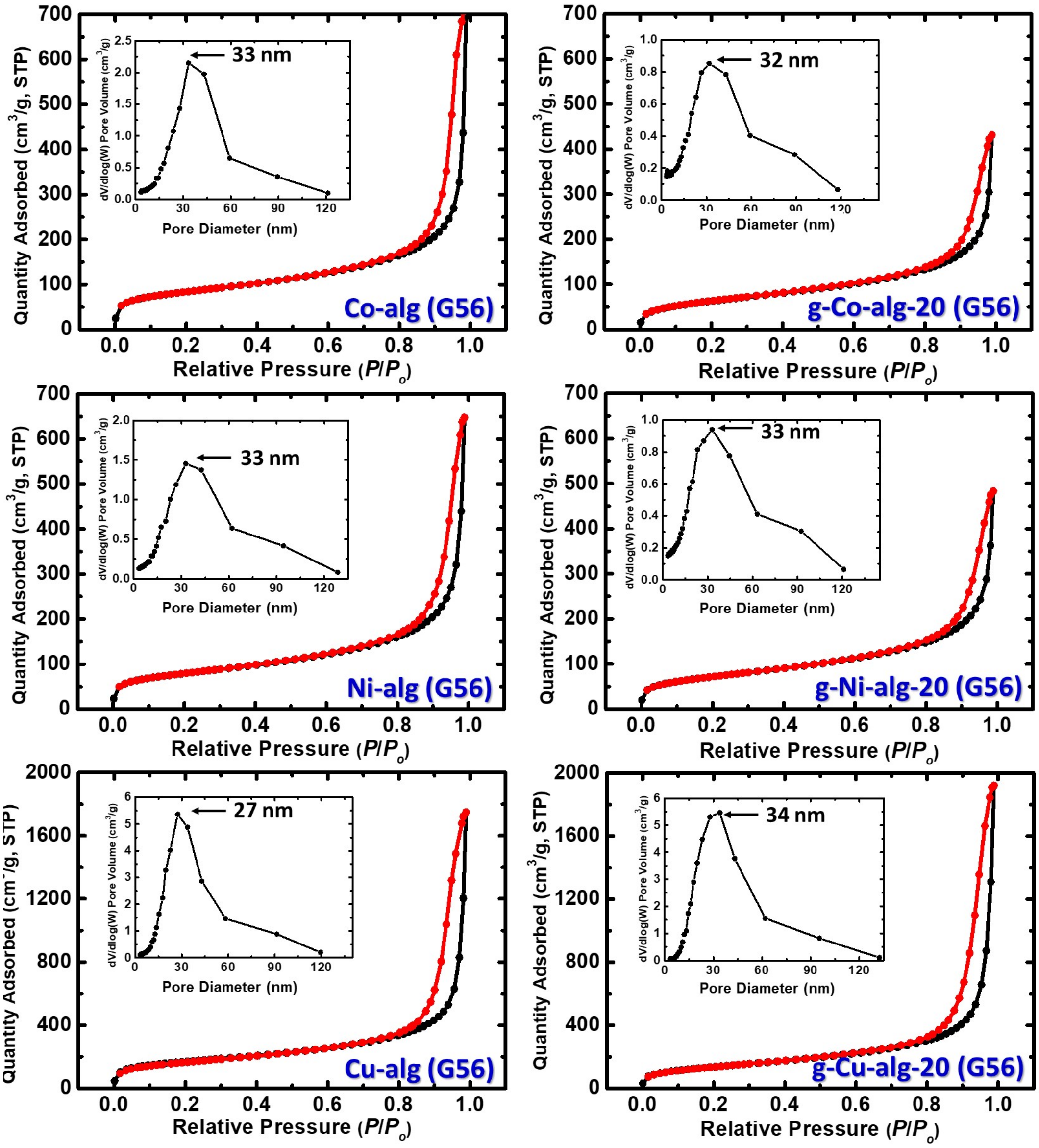

Figure S9. N_2-sorption diagrams of M-alginate and g-M-alginate (G56) aerogels, as indicated. Insets show pore size distributions by the BJH method.
